# 

**Published:** 1906-07-15

**Authors:** 


### ANNOUNCEMENTS.

WILL STAY OUT OF POLITICS.—The members of the Iowa State Dental Society passed a resolution at their meeting at Des Moines that in the future they will refuse to electioneer for candidates for the State Dental Board, or to take an active part in the contests.—*Iowa City Republican.*

FIRST ANNUAL REPORT ROCHESTER FREE DENTAL DISPENSARY.—On Washington's birthday, February 22, 1905, the Rochester Dental Society, under the auspices of the Rochester Public Health Association, opened a Free Dental Dispensary at 32 South Washington Street, for the care of the teeth of the worthy poor.

Equipment and materials valued at $1,200 were installed; one-half of this amount was donated in cash by a member of the Rochester Public Health Association and one-half by members of the Dental Society, City Merchants and Dental Manufacturers and Dealers throughout the world.

During the first year, twenty-four members of the Society alternated in attendance at the Dispensary.

January 15, 1906, an operating staff of three dentists were appointed; one of whom is in attendance from 2 to 5 P. M. each week-day.

Report of operations to March 1, 1906, are as follows: Patients treated, 203; Separate visits made, 478; Operations performed, 1078. F. W. PROSEUS,

*Chairman Committee.*

---

A NEW POST GRADUATE SCHOOL.—Drs. Nevins, Green and Ream, of Chicago, have launched a school for instruction in dental anesthesia and the extraction of teeth. There can be little question but that such instruction is sadly needed, and we trust the enterprise may prosper.

---

A PUBLIC DENTAL LIBRARY.—The library board of the new Carnegie Library, Columbus, O., has consented to give space to the dentists for a dental library and reading room. Here is an opportunity to build one of the best dental libraries in the country, and not alone for Columbus dentists, but for the benefit of dentists generally throughout the state, as everyone will be welcome. All the dental literature possible will be kept in the library, making it one of great value for reference.

Columbus being centrally located and easy of access by so many dentists, it would seem to be an ideal location for a general dental library.

The building is still under construction, but will be ready for occupancy by fall, and already the dental committee is looking about to ascertain what may be depended on in the way of contributions of books and journals. Every book contributed will be inscribed with the name of the donor, and it will remain thus recorded in the library.

If any of our readers have books or journals they would like to contribute, they will confer a favor by sending titles to either Dr. W. H. Todd or Dr. E. C. Mills, Columbus, O., members of the dental library committee.

The above is taken from the June issue of the *Dental Summary*, and we desire to most cordially endorse the idea. There should be a first-class dental library in every state, and this, it seems to us, is an ideal opportunity for the dentists of Ohio to gather under the most favorable conditions a good working library which will be available for any dentist who may wish to consult it. In the years to come it will be more appreciated than it is likely to be now.

—✦—

THE AMERICAN DENTAL SOCIETY OF EUROPE will meet in Berlin, Germany, August 1–4. All ethical Americans traveling in Europe are invited to attend.

—✦—

FEDERATION DENTAIRE INTERNATIONALE.—The next meeting of this body will be held at Geneva, Switzerland, August 8th and 9th. This organization is doing a good work although there has not as yet come any satisfactory results from its consideration of our International educational problems. It furnishes the only place for a consideration of the questions by the leaders of thought of the various countries and it would seem that eventually tangible results ought to result. The best in the educational problem ought to be developed from the exchange of ideals and methods.

—✦—

THE SWISS ODONTOLOGICAL SOCIETY will hold its annual meeting immediately following the Federation meetings and a cordial invitation is extended to all who can be present to attend both meetings, as great efforts will be made to entertain all visitors.

—✦—

Missouri State Dental Association.—The forty-first annual meeting of the Missouri State Dental Association was held June 5, 6, and 7, at Springfield, Mo. An important feature of the meeting was the adoption of a plan for organizing societies throughout the State. The following officers and committees were elected: President, F. G. Worthley, Kansas City; Corresponding Secretary, E. P. Dameron, St. Louis; Secreatry, J. T. Fry, Moberly. The next meeting will be held in Kansas City.

—✦—

Columbus, Ohio, Dental Society.—At its last meeting the following officers were elected for the year: President, H. C. Brown; Vice-President, L. P. Bethel; Secretary, R. R. Smith; Treasurer, W. B. Kiger. With such a set of officers that society ought to make things interesting.

—✦—

South Dakota Examining Board.—The next meeting of this board will be held July 31st, 1906, at Sioux Falls. For full particulars address

Dr. G. W. Collins, *Secretary*,
Vermillion, South Dakota.

—✦—

New York State Dental Society.—At the session of the Dental Society of the State of New York $700 was raised for the relief of the San Francisco sufferers. The award of gold medals, from the fund donated by Dr. Wm. Jarvie last year, to be awarded to men who have made meritorious scientific research during the year of value to dentistry was made. Three metals were awarded and the following were the men honored: Dr. G. V. Black, of Chicago; Dr. E. T. Darby, of Philadelphia and Dr. W. D. Miller, of Berlin, Germany, an American, sojourning there. The medals were of most beautiful design, almost square in shape and arranged to be worn as watchcharm or fob. Officers elected for the ensuing year are as follows: President, Dr. W. A. White, Phelps; Vice-president, Dr. W. S. Rose, Schenectady; Secretary, Dr. C. S. Butler, Buffalo; Treasurer, Dr. C. W. Stainton, Buffalo; Correspondent, Dr. S. L. Goldsmith, New York. The president-elect was empowered to appoint delegates to the National Dental Association.

—✦—

National Dental Association.—The tenth annual session of the National Dental Association will be held in Atlanta, Ga., commencing Tuesday, September, 18, 1906. The New Kimball House has been selected by the local committee of Arrangements as headquarters where all general sessions of the association and of the sections will be held. The rates per day at the New Kimball House will be, European Plan, from $1.50 to $4.00; American Plan, from $3.00 to $6.00, governed by choice of rooms. The usual railroad rate of one and one-third fare for the round trip, certificate plan, will be arranged for, and definite dates and particulars given later, by Dr. J. D. Patterson, Chairman of the Executive Committee.

—✦—

Golden Anniversary Banquet.—To commemorate the fiftieth annual session of the Pennsylvania College of Dental Surgery a golden aniversary banquet was held under the auspices of the Alumni Association, May 31, 1906, at the Hotel Bellevue-Stratford, Philadelphia. Four of the five surviving members of the class of 1854, Philadelphia College of Dental Surgery, were present as guests. Dr. C. N. Pierce, president of the Alumni Association, presided, and Dr. Wilbur S. Litch, acted as toastmaster. The following toasts were responded to:

"The Pennsylvania College of Dental Surgery," Dr. William H. Trueman; "The Board of Corporators," Dr. I. Minis Hays; "The Faculty," Dr. George W. Warren; "The Class of 1854, Philadelphia College of Dental Surgery," Dr. James Trueman; "The Dental Council of Pennsylvania," Dr. Nathan C. Schaeffer; "The Alumni," Dr. Edward C. Kirk; "The Auxiliary Instructors," Dr. Frank G. Ritter.—*Brief.*

—✦—

FREE DENTAL SERVICE FOR THE POOR.—The Milton Pennsylvania Dental Association has been guilty of a deed of charity that everybody should know. It has agreed to perform work for the poor children of the town without cost, devoting all day Friday of each week to the good cause. The boys and girls must be recommended by the several school teachers of the town and a blank has been printed which reads: "Mr. Blank of Blank Street, is recommended to the Milton Dental Association for treatment; Miss Blank, teacher." This has the endorsement of Prof. Wilson, of the Board of Control, the teachers, and the work will be done in the same style as is done for pay by the more fortunate ones. For this month Dr. Krumrine will officiate and in turns of a month each they will alternate until the doctor again is called upon to do good.—*Milton Miltonian.*

—✦—

NO PORTRAITS AND FREE DENTAL TREATMENT OF THE POOR.—The Susquehanna Dental Society at its forty-third annual meeting in Allentown, Pa., added to its code of ethics a rule to prohibit members from furnishing portraits for publications in the newspapers as conduct unbecoming the profession. It was also decided to establish in each county one place for free dental treatment of the poor.—*Brief.*

